# Enrichment of schizophrenia heritability in both neuronal and glia cell regulatory elements

**DOI:** 10.1038/s41398-017-0053-y

**Published:** 2018-01-10

**Authors:** Katherine E. Tansey, Matthew J. Hill

**Affiliations:** 1 Core Bioinformatics and Statistics Team, College of Biomedical and Life Sciences, Cardiff University, Cardiff, UK; 2 MRC Centre for Neuropsychiatric Genetics and Genomics, School of Medicine, Cardiff University, Cardiff, UK; 3 Neuroscience and Mental Health Research Institute, School of Medicine, Cardiff University, Cardiff, UK

## Abstract

Genome-wide association studies have identified over 100 robust risk loci for schizophrenia with thousands of variants mediating genetic heritability, the majority of which reside in non-coding regions. Analytical approaches have shown this heritability is strongly enriched at variants within regulatory elements identified from human post-mortem brain tissue. However, bulk post-mortem brain tissue has a heterogeneous cell composition, making biological interpretations difficult. We sought to refine the cell types mediating schizophrenia heritability by separating neuronal and glial signals using data from: (1) NeuN-sorted post-mortem brain and (2) cell culture systems. Schizophrenia heritability was partitioned using linkage disequilbrium (LD) score regression. Variants within genomic regions marked by H3K4me3 (marker of active promoters) from NeuN-positive (neuronal) and NeuN-negative (non-neuronal) cells explained a significant amount of schizophrenia heritability (*P* = 1.38 × 10^−10^ and *P* = 7.97 × 10^−10^). However, variants located in H3K4me3 sites specific to NeuN-positive (neuronal) cells were enriched (*P* = 3.13 × 10^−4^), while those specific to NeuN-negative (non-neuronal) cells were not (*P* = 0.470). Data from cell culture systems mimicked this pattern of association. We show the previously observed enrichment of heritability from variants at brain H3K4me3 sites is mediated by both neuronal and non-neuronal brain cell types. However, only neuronal cell populations showed a unique contribution driven by cell-type specific regulatory elements. Cell culture systems recapitulate disease relevant gene-regulatory landscapes, validating them as a tool for future investigation of genetic mechanisms underlying schizophrenia. Identifying the cell types in which risk variants operate will greatly increase our understanding of schizophrenia pathobiology and aid in the development of novel model systems and therapies.

## Introduction

Genome-wide association studies (GWAS) have successfully identified genetic loci mediating human diseases and phenotypes. For schizophrenia, more than 100 genome-wide significant loci have been identified^1^. Although individual variants weakly influence disease risk, collectively common variants, including those that do not yet reach statistical significance, explain a substantial proportion of the heritability of schizophrenia^2,3^. Consistent with the genetic architecture of other common complex disorders, the majority of risk variants are located within non-coding regions of the genome, suggesting that they alter risk by changing levels of gene expression and/or splicing. Mechanistically, causal variants will disrupt the function of non-coding regulatory elements in cell types relevant to disease. Similarly, although the heritability of complex diseases is governed by thousands of variants across the genome, it is enriched at regulatory elements from specific cell types^4^. These measurements of partitioned heritability, as well as other enrichment analyses^5^, can provide powerful insights into the tissue and cell types underlying pathobiology, which are important for establishing valid disease models and assays of gene function.

Several large-scale projects (e.g. ENCODE^6^, Epigenetics Roadmap^7^, BLUEPRINT^8^) have begun to map the regulatory landscape of different human cell and tissue types. Using these data it has been shown that schizophrenia heritability is most strongly enriched at variants within regulatory regions identified in post-mortem brain tissue^4^, particularly those marked by the histone modification H3K4me3, a modification enriched at active gene promoters^9^. However, the brain is composed of many different cells types, making biological interpretation of these results difficult. A fuller understanding of disease risk mechanisms will require detailed analysis of specific cell types, developmental stages and physiological contexts.

The broadest subdivision of brain cell types is the separation of neurons and glia. We sought to investigate the relative importance of these two major cell populations in mediating schizophrenia genetic risk. Given that a large number of variants influence genetic risk for schizophrenia, including those outside genome-wide significant loci, we selected to use measurements of heritability that incorporate genome-wide data. Firstly, we partitioned the heritability of schizophrenia from the latest GWAS available from the Psychiatric Genomics Consortium using epigenomic data from NeuN-sorted post-mortem brain cells, and secondly, using epigenomic data from cultured human neurons and astrocytes. In doing so, we also investigated the validity of cell culture systems, including human stem cell-derived neurons, as tools for understanding schizophrenia genetic risk mechanisms.

## Methods

### Chromatin immunoprecipitation-sequencing data sets and processing

H3K4me3 chromatin immunoprecipitation (ChIP)-sequencing data from human embryonic stem cell-derived neurons were generated by the Epigenomics Roadmap^7^. Preprocessed broad peak calls for H3K4me3 were downloaded from http://egg2.wustl.edu/roadmap/data/byFileType/peaks/consolidated/broadPeak/. Peaks with a negative log10 *P*-value of 5 or greater were retained for further analysis, as described in the original MACS protocol^10^.

H3K4me3 ChIP-sequencing data from NeuN-positive and NeuN-sorted human post-mortem brain nuclei^11^ (GEO accession numbers: GSM529964, GSM529969, GSM529971 and GSM529972) were downloaded from the Sequence Read Archive (SRA, accession number: GSE21172). These data correspond to chromatin immunoprecipitation (ChIP) experiments performed on two donor samples, for which both NeuN-positive and NeuN-negative sorted cell populations were available. SRA files were converted to fastq files using fastq-dump, part of the SRA toolkit available from NCBI (http://www.ncbi.nlm.nih.gov/Traces/sra). Fastq files were then processed as previously described^12^. Briefly, data were aligned using bowtie2^13^, and converted to bam, sorted and indexed using SAMtools^14^. The resulting bam files for two biological replicates for each cell type were merged using BEDTools^15^ to produce single consolidated read sets for NeuN-positive and NeuN-negative cell types. All four files (two biological replicates for each cell type) were also merged to produce an additional read set that mimicked data generated from non-sorted post-mortem tissue, hereafter called ‘reconstituted’ brain data set. Peaks were called as previously described^12^ using broad peak calling in MACS 2.0^16^ with broad-cutoff = 0.1 and *q-*value = 0.01.

Aligned H3K3me3 ChIP-seq reads generated from astrocytes^6^ were downloaded from GEO (GSM733747) along with matched input samples (GSM733678). Peaks were called for each replicate as described above. Peaks with a negative log10 *P*-value of 5 or greater were retained and the resulting peak files intersected using BEDTools^15^ produce a peak set used in further analysis.

H3K4me3 and input ChIP-sequencing data from human adipocyte cells were downloaded from GEO series GSE41629 and processed as described for the astrocytes, with the exception that these data only consisted of a single replicate. This peak set was used as a negative control in the analysis to ensure results were specific to the brain cell types of interest.

Cell-type specific peaks were extracted by intersecting the appropriate processed bed files (e.g. NeuN-positive peak file and NeuN-negative peak file) using BEDTools^15^.

### GWAS summary statistics

Schizophrenia GWAS summary statistics were download from https://www.med.unc.edu/pgc/results-and-downloads. Data used were from the largest schizophrenia GWAS undertaken to date that is publicly available, the Psychiatric Genomics Consortium’s 2014 study (PGC-SCZ)^1^. The PGC-SCZ study included 79,845 individuals with a case control ratio of 0.429.

### Partitioning heritability

Heritability was partitioned using LD score regression^3^ following previously described methodology^4^. Consistent with previous partitioning heritability studies^4^, all peaks were extended by ±500 base pairs. LD score files were made for each specific annotation of interest using the open source software available here: https://github.com/bulik/ldsc/wiki. The major histocompatibility complex (MHC) region was removed from the schizophrenia GWAS summary statistics as was done in the original analysis due to the LD structure of the region. Each annotation was added to the baseline model independently creating 12 separate models, as was done in the original analysis^4^. The baseline model includes 24 non-cell-specific annotations that cover a range of DNA features, such as coding, three prime untranslated region, promoter, intronic, H3K4me1 marks, H3K4me3 marks, H3K9ac marks, H3K27ac marks, DNase I hypersensitivity sites, chromHMM and Segway predictions, regions conserved in mammals, super-enhancers, and FANTOM5 enhancers (please see ref. 4 for more information about the baseline model). *Z*-scores were extracted from each model for the annotation of interest and were used to calculate *P*-values, hereafter called *z*-score *P*-values. Additionally, we performed a conditional partitioned heritability analysis. For this, we ran a model that included both NeuN-positive and NeuN-negative H3K4me3 annotations along with the baseline, and another model that included human stem cell-derived neurons and cultured primary human astrocytes H3K4me3 annotations along with the baseline.

We report the enrichment of schizophrenia heritability within each cell-type specific functional category, as enrichment is more easily understood and interpretable in terms of the relationship between schizophrenia heritability and cell-type specific functional categories than *z*-scores. However, we report the *z*-score *P*-value alongside these enrichments instead of the enrichment *P*-value as *z*-score *P*-value controls for the overlap of cell-type-specific annotations with other functional categories that are included in the full baseline model^4^ or annotations included in a conditional partitioned heritability analysis, whereas enrichment *P*-values do not.

In total, we performed 12 tests of enrichment and two conditional tests for a total of 14 tests. Using Bonferroni’s correction for multiple testing, *P*-values below *P* < 3.57 × 10^−3^ were considered to have withstood correction for multiple testing.

## Results

**Fig. 1:**
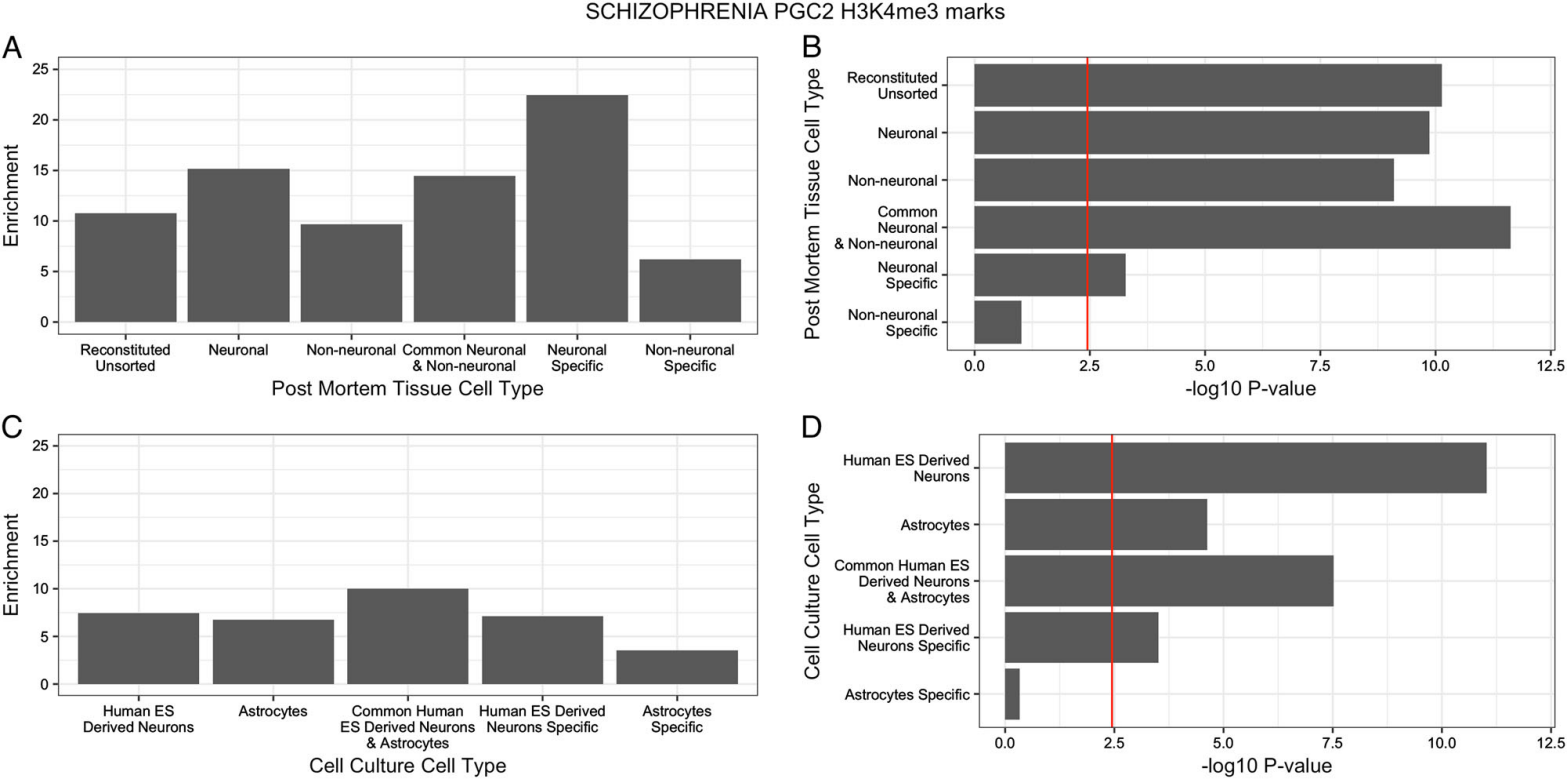
Enrichment and −log10 *P*-value for the results of partitioned heritability analysis for SNPs within H3K4me3 marks (a marker of active gene promoters) for the different cell types . **a** Enrichment of schizophrenia heritability at H3K4me3 sites in post-mortem brain tissues. *Y*-axis is the enrichment value and *x*-axis is the different cell types. **b** −log10 *P*-values for the enrichment of schizophrenia heritability at H3K4me3 sites in post-mortem brain tissues. *X*-axis is the −log10 *P*-value derived from the *z*-score; *y*-axis is the different cell types. Red line is the significance threshold for a Bonferroni correction for multiple testing for total number of analyses undertaken (14 tests performed). **c** Enrichment of schizophrenia heritability at H3K4me3 sites identified in cultured cells. *Y*-axis is the enrichment value and *x*-axis is the different cell types. **d** −log10 *P*-values for enrichment of schizophrenia heritability at H3K4me3 sites identified in cultured cells. *X*-axis is the −log10 *P*-value derived from the *z*-score; *y*-axis is the different cell types Red line is the significance threshold for a Bonferroni correction for multiple testing for total number of analyses undertaken (14 tests performed)

To confirm the previously reported enrichment of heritability at variants located in brain H3K4me3 sites^4^, and to validate the post-mortem brain data used here, we first combined the NeuN-positive and NeuN-negative H3K4me3 data to produce a ‘reconstituted’ brain data set. We observed a significant enrichment of heritability at variants located in these regions (enrichment (SE) = 10.789 (1.221); *z*-score *P* = 7.37 × 10^−11^; Table 1; Fig. 1), with a similar magnitude and significance as previously reported for unsorted brain tissue^4^, confirming the suitability of these data for further analysis.

**Table 1:** Results from the partitioned heritability analysis for the cell types tested for enrichment of SNPs within H3K4me3 marks (a marker of active gene promoters)

Cell type	Proportion of SNPs	Proportion of *h* ^2^ (SE)	Enrichment (SE)	Enrichment *P*-value	*Z*-score *P*-value
‘Reconstituted’ brain (NeuN positive and NeuN negative)	0.021	0.225 (0.025)	10.789 (1.221)	3.13 × 10^−13^	**7.37 × 10** ^**−11**^
Neuronal (NeuN positive)	0.013	0.195 (0.024)	15.178 (1.848)	2.99 × 10^−12^	**1.38 × 10** ^**−10**^
Non-neuronal (NeuN negative)	0.018	0.178 (0.023)	9.674 (1.225)	1.82 × 10^−11^	**7.97 × 10** ^**−10**^
Common neuronal and non-neuronal (NeuN positive and NeuN negative)	0.010	0.150 (0.019)	14.455 (1.820)	3.00 × 10^−12^	**2.39 × 10** ^**−12**^
Neuronal specific (NeuN-positive only)	0.002	0.054 (0.013)	22.476 (5.470)	1.87 × 10^−4^	**5.34 × 10** ^**−4**^
Non-neuronal specific (NeuN negative only)	0.008	0.050 (0.016)	6.199 (1.961)	8.43 × 10^−3^	0.097
*Conditional analysis* Neuronal (NeuN positive)	0.013	0.195	15.214	2.55 × 10^−12^	**3.56 × 10** ^**−6**^
		(0.024)	(1.847)		
*Conditional analysis* Non-neuronal (NeuN negative)	0.018	0.189 (0.023)	10.291 (1.235)	1.41 × 10^−12^	0.102
Human ES-derived neurons	0.021	0.159 (0.018)	7.436 (0.839)	7.19 × 10^−13^	**9.52 × 10** ^**−12**^
Astrocytes	0.017	0.118 (0.018)	6.766 (1.048)	9.68 × 10^−8^	**2.37 × 10** ^**−5**^
Common human ES-derived neurons and astrocyte	0.010	0.105 (0.015)	9.992 (1.393)	7.81 × 10^−10^	**3.00 × 10** ^**−8**^
Human ES-derived neurons specific	0.011	0.077 (0.015)	7.122 (1.393)	1.66 × 10^−5^	**3.13 × 10** ^**−4**^
Astrocytes specific	0.007	0.024 (0.012)	3.514 (1.749)	0.151	0.470
*Conditional analysis* Human ES-derived neurons	0.021	0.159 (0.018)	7.476 (0.838)	5.32 × 10^−13^	4**.72 × 10** ^**−8**^
*Conditional analysis* Astrocytes	0.017	0.112 (0.018)	6.441 (1.054)	5.15 × 10^−7^	0.043
Adipocytes	0.025	0.092 (0.023)	3.691 (0.918)	3.73 × 10^−3^	0.070

*h* ^2^ is the genetic heritability. SE is the standard error. *Z*-score *P*-values below the Bonferroni corrected *P*-value of *P* < 3.57 × 10^−3^ are shown in bold. ES, embryonic stem

Next we used variants in H3K4me3 sites identified in NeuN-positive and NeuN-negative cells separately to investigate the relative contribution of each cell type to schizophrenia risk. There was significant enrichment of heritability from variants located in H3K4me3 sites from both cell populations (NeuN-positive enrichment (SE) = 15.178 (1.848); *z*-score *P* = 1.38 × 10^−10^; NeuN-negative enrichment (SE) = 9.674 (1.225); *z*-score *P* = 7.97 × 10^−10^; Table 1; Fig. 1).

Given an enrichment of heritability was observed from variants located in H3K4me3 sites in either cell population, we investigated the contribution of variants located in common and cell-type specific H3K4me3 sites. SNPs in H3K4me3 marked regions that were shared between neuronal (NeuN positive) and non-neuronal (NeuN-negative) brain cells showed the most significant enrichment (enrichment (SE) = 14.455 (1.820); *z*-score *P* = 2.39 × 10^−12^; Table 1; Fig. 1). However, a high level of enrichment was also observed at SNPs located in H3K4me3 regions specific to neuronal cells (NeuN positive) (enrichment (SE) = 22.476 (5.470); *z*-score *P* = 5.34 × 10^−4^; Table 1; Fig. 1). In contrast, SNPs in non-neuronal (NeuN negative) cell-specific H3K4me3 elements showed no significant enrichment (enrichment (SE) = 6.199 (1.961); *z*-score *P* = 0.097). A conditional partitioned heritability analysis also confirmed these results with H3K4me3 regions in neuronal cells (NeuN positive) remaining significantly associated (*z*-score *P* = 3.60 × 10^−6^) when H3K4me3 regions in non-neuronal cells (NeuN negative) was included in the model, and H3K4me3 regions in non-neuronal cells (NeuN negative) did not remain associated (*z*-score *P* = 0.102) (Table 1).

This pattern of enrichment was replicated in cell culture systems using human stem cell-derived neurons and cultured primary human astrocytes. Consistent with the results from sorted post-mortem brain, enrichment of heritability was observed from SNPs located in neuronal (enrichment (SE) = 7.436 (0.839); *z*-score *P* = 9.52 × 10^−12^) and non-neuronal (enrichment (SE) = 6.766 (1.048); *z*-score *P* = 2.37 × 10^−5^) H3K4me3 sites (Table 1; Fig. 1). SNPs residing in H3K4me3 elements shared by human stem cell-derived neurons and astrocytes were also enriched (enrichment (SE) = 9.992 (1.393); *z*-score *P* = 3.00 × 10^−8^; Table 1; Fig. 1). In agreement with the post-mortem cell-type-specific elements, SNPs residing in H3K4me3 sites specific to human stem cell-derived neurons were also significantly enriched (enrichment (SE) = 7.122 (1.393); *z*-score *P* = 3.13 × 10^−4^), while those SNPs within H3K4me3 elements specific to astrocytes were not (enrichment (SE) = 3.514 (1.749); *z*-score *P* = 0.470) (Table 1; Fig. 1). Performing a conditional partitioned heritability analysis resulted in a similar association pattern with H3K4me3 regions in human stem cell-derived neurons remaining significantly associated (*z*-score *P* = 4.72 × 10^−8^) when H3K4me3 regions in cultured primary human astrocytes were included in the model, and H3K4me3 regions in cultured primary human astrocytes were only weakly associated (*z*-score *P* = 0.043) (Table 1).

No significant enrichment at SNPs located in H3K4me3 sites identified in adipocytes, a negative control tissue, was observed (enrichment (SE) = 3.691 (0.918); *z*-score *P* = 0.070; Table 1), indicating enrichment specific to brain cell types tested.

## Discussion

Our analysis sought to investigate the relative contribution of two major brain cell types, neurons and glia, to genetic risk mechanisms for schizophrenia. Using measurements of genome-wide partitioned heritability, we find that both neurons and glia are potential mediators of genetic risk for schizophrenia. This finding is robust across two distinct methods of cell separation, neuronal cell sorting of post-mortem tissue and cultured cells. However, variants in neuronal H3K4me3 marked regions remained enriched after accounting for the contribution of non-neuronal cells, indicating that the neuronal cell populations may be more important. This pattern is also consistent when considering cell-specific H3K4me3 marks; an enrichment in SNPs located within neuronal specific but not non-neuronal H3K4me3 marks is observed when using both post-mortem tissue and cultured cells. The contribution of non-neuronal brain cell types to schizophrenia pathology has been documented previously^17,18^, but their role in primary pathobiological mechanisms was unknown. Our results suggest that genetic risk for schizophrenia plausibly acts, in part, via direct disruption of glial cell function. However, common genetic variant schizophrenia heritability is unlikely to be mediated by regulatory elements that are specific to glial cells, but instead operate via regulatory elements that are common to both neurons and glia, or those that are specific to neurons. Nevertheless, subsequent biological investigations should consider the role of both neurons and glia in schizophrenia pathobiology and treatment.

Although genomic studies of schizophrenia have identified robust risk loci, major challenges exist when attempting to translate these findings into biological mechanisms. For non-coding variation, which includes the majority of risk variants discovered by GWAS, this includes identifying the tissue/cell types in which the genetic variants exert their effect^19^. The tissue specificity of non-coding elements, and by extension the effects of variants in these elements, is defined by post-translational modification of histones, chromatin structure and transcription factor binding. The integration of genomic findings with detailed maps of gene-regulatory elements can therefore empirically determine the tissue and cells types most relevant for disease risk. Similarly, integration with expression and methylation quantitative trait loci has been used to implicate brain active regulatory variants in psychiatric disease risk mechanisms^20,21^. However, tissues are comprised of a complex mix of cells, each with distinct gene expression and regulatory DNA element repertoires.

The complex cellular composition of the human brain is therefore a significant barrier to the interpretation of non-coding variant-mediated genetic mechanisms of neuropsychiatric and neurodegenerative diseases. Without detailed cell-type information our ability to produce valid disease models and novel therapies is hampered. Although our findings suggest that both neurons and glia are potential mediators of genetic risk, it will be important to investigate the contribution of cells types at a much higher resolution than current data provided (e.g. individual neuronal subtypes). Importantly, we show that the use of sorted nuclei from post-mortem material and cell-type-specific culture models are both likely to be useful approaches for investigating the contribution of defined cell types to genetic risk mechanisms of schizophrenia.

Although we see strong agreement between results from post-mortem tissue and in vitro systems, they do not correspond to identical cell populations. Specifically, the sorted post-mortem cell populations are likely to be more heterogeneous than their cell culture counterparts. For example, although the NeuN-negative cell population is largely composed of astrocytes, mimicking the primary cell culture system included in our analysis, it will also contain other NeuN-negative cell types (e.g. microglia and oligodendrocytes). Similarly, the NeuN-positive cell population contains a mixture of neuronal subtypes that are not likely be captured in the single-cell culture model used here. These differences in cell composition, along with other factors (e.g. immaturity of stem cell-derived neurons), may underlie the larger enrichments observed when using post-mortem brain tissue compared with cell culture systems.

Improved methods for the selective isolation of cell populations from post-mortem brain tissue^22^, along with directed differentiation of human stem cells, will be powerful tools for investigating the regulatory landscape of human brain cells. Subsequent integration with disease genomics promises to identify specific risk loci acting in defined cells types that will aid in our biological understanding of disease risk mechanisms.

In summary, using measurements of partitioned heritability, we show that common variant schizophrenia heritability is significantly enriched within H3K4me3 marked regions identified in neuronal and non-neuronal cell populations of the human post-mortem brain. This finding is replicated using data from tissue culture systems, where cell types can be differentiated and grown in isolation. Together, these results show that schizophrenia genetic risk mechanisms potentially operate in both neuronal and non-neuronal cells of the brain. However, the greatest enrichment arises from the neuronal cell population, as well as from neuronal-specific regulatory elements. Strong agreement between results from post-mortem cell types and cell culture systems validate in vitro systems as tools for studying genetic risk mechanisms of schizophrenia. Future investigations of gene-regulatory landscapes using defined cell types, either from post-mortem tissue or from in vitro models, will further our understanding of schizophrenia genetic risk mechanisms.
